# Design and validation of an instrument to evaluate Person-Centered care in health services

**DOI:** 10.1186/s13690-024-01324-2

**Published:** 2024-08-14

**Authors:** Rodolfo Lehmann-Mendoza, Gabriela Yanet Cortés-Moreno, Odet Sarabia-González, Rosa Paola Figuerola-Escoto, David Luna, Ilicia González-Mundo, Rubén Caselín-Ledezma, Roberto Arturo Vázquez-Dávila, Hilario Ascención Martínez-Arredondo

**Affiliations:** 1 Subdirección de Servicios de Salud de Petróleos Mexicanos, Ciudad de México, México; 2 Coordinación Nacional de Investigación, Petróleos Mexicanos, Ciudad de México, México; 3 Subgerencia de Calidad, Servicios de Salud de Petróleos Mexicanos, Ciudad de México, México; 4 Centro Interdisciplinario de Ciencias de Salud, Instituto Politécnico Nacional, Ciudad de México, México; 5 Unidad de Investigación Multidisciplinaria en Salud, Instituto Nacional de Rehabilitación Luis Guillermo Ibarra Ibarra, Ciudad de México, México; 6 Universidad Popular Autónoma del Estado de Puebla, Puebla, México; 7 Subgerencia de Educación y Desarrollo en Salud, Petróleos Mexicanos, Ciudad de México, México; 8 Gerencia de Medicina Preventiva, Petróleos Mexicanos, Ciudad de México, México

**Keywords:** Person-centered care, Instrument design, Instrument validation, Exploratory factorial analysis, Confirmatory factorial analysis

## Abstract

**Background:**

The concept of quality in health care has evolved, placing greater importance on the patient’s needs, culture, and social context, as well as their participation in clinical decision-making, as highlighted by Mead and Bower’s Person-Centered Care Model. The aim of the present study was to design and validate an instrument to assess the extent to which healthcare services provided by PEMEX (Petróleos Mexicanos) offer person-centered care according to user perceptions.

**Methods:**

The first phase comprised the development of 57 items based on the analysis of responses from an open-ended questionnaire administered to 30 users of Pemex healthcare services. This questionnaire was designed considering the four factors of the person-centered care model, however, the high correlation between the 4 factors (i.e., *r* ≥ .80) indicated an overfactoring effect and consequently an increase in the risk of overfitting. Therefore, an exhaustive analysis of the instrument was performed, starting with the review of the individual behavior of each item, and carrying out exploratory and confirmatory factor analysis. Using a sample of 330 individuals, an exploratory factor analysis was perfomed. Afterward, a confirmatory factorial analysis was carried out with 335 participants. Finally, a new confirmatory factorial analysis included 130 participants due to the refinements made in the previous phase. Internal consistency was assessed using Cronbach’s α and McDonald’s ω at every phase.

**Results:**

The exploratory factor analysis retained 35 items in a single factor that accounted for 49% of the variance with an internal consistency of Cronbach’s α and McDonald’s ω = 0.97. Because the factorial structure by confirmatory factorial analysis was unsatisfactory, the initial model was refined, leading to the retention of 11 items and a final model with adjustment index of χ2 = 127.53, χ2/gl = 2.89, RMSEA = 0.07, IC RMSEA 0.06 to 0.09, TLI = 0.95 and CFI = 0.96, with an internal consistency of Cronbach’s α and McDonald’s ω = 0.93. Due to the refinements, a new confirmatory factorial analysis was conducted with suitable goodness-of-fit criteria in most items (χ2 = 151.44, χ2/gl = 3.43, RMSEA = 0.13, IC RMSEA 0.11 to 0.16, TLI = 0.93 and CFI = 0.94), resulting in a Cronbach’s α and McDonald’s ω = 0.98.

**Conclusions:**

The instrument exhibits suitable psychometric properties to be employed to measure the degree to which medical care is patient centered. This instrument represents a strategy for promoting an innovative healthcare model.

**Supplementary Information:**

The online version contains supplementary material available at 10.1186/s13690-024-01324-2.

**Table:** 

**Text box 1. Contributions to the literature**
•Patient-centered care is about seeing everyone as a unique human being, with needs, desires, and concerns of their own. It means listening carefully, showing empathy, and working collaboratively with our patients to provide them with the best care possible. The creation of an instrument that measures patient-centered care, will detect the need to empower patients to improve results related to health care and increase the medical services user´s satisfaction.
•This innovative instrument was built from all the factors contemplated in patient-centered care: (1) biopsychosocial perspective, (2) patient as a person, (3) therapeutic alliance, and (4) sharing power and responsibilities, and although the statistical treatment resulted in a unifactorial instrument, by conserving items that initially represented 3 of the 4 fundamental factors of the theoretical model, we consider that it preserves the essence of the theoretical model proposed by Mead and Bower [4].
•The strength of having designed an instrument whose rationale, design and validation are based on the highest scientific rigor by mean of a robust statistical analyses, such as exploratory factor analysis to detect its structure and confirmatory factor analysis to validate such structure is fundamental to the public health literature.

## Introduction

The organization of healthcare service components aims to contribute to the collective function. In this sense, the analytical and descriptive representation of a series of goals, operational strategies, and healthcare objectives that meet the population needs and demands allows conceptualizing a healthcare delivery model. Drawing from the insights of Avedis Donabedian [1], the extent to which healthcare services provide the greatest benefit, with minimum risks and reasonable costs, will be proportional to the quality of healthcare delivered.

The concept of quality in healthcare applied to the healthcare sector has been evolving, primarily in terms of the patient‒doctor relationship, where the principle of autonomy has been established gradually. This principle emphasizes the patient as a decision-maker regarding their health status [2]. Prior to this, there was a prevailing approach in which doctors played an exclusive role in decision-making and action guidelines. Currently, healthcare professionals strive to incorporate patient´s preferences into decisions regarding their care and engage in shared decision-making between the patient and the healthcare provider. The goal is to involve those affected by clinical decision-making [3].

The involvement of the patient in their health care and clinical decision-making highlights the “person-centered” care model by Mead & Bower, which focuses on the approach and understanding of patient´s experience and the meaning that illness holds for them. This model comprises four dimensions: (1) biopsychosocial perspective, (2) patient as a person, (3) therapeutic alliance, and (4) sharing power and responsibilities [4]. Identifying and understanding the patient’s perspective enables their inclusion in decision-making and care evolution [4]. While this approach has been discussed since the 1980s, it was not until the 1990s that it gained recognition and began to be implemented with the aim of shaping healthcare delivery centered on patients rather than providers and health systems [5]. Providing person-centered care involves respecting the patients´ culture, social context, and needs. In addition, it expects the patient’s role in care to be active, participating in decisions around their health care [6]. Empowering the patient encourages a responsible attitude toward their health care and brings advantages compared to other care models [7–11].

If healthcare aims to provide person-centered care, it is then essential to understand quality from the patient´s perspective [12, 13], whose perception encompasses subjective and objective experiences and observations of healthcare staff behavior influenced by different variables. Therefore, the patient’s experience must be considered a reflection of the commitment adopted by healthcare providers and the quality of care [14]. Definitively, factors such as the healthcare system’s reputation, concern for health status, and previous interactions with the medical unit impact patient expectations and consequently the perception of healthcare [13].

Regarding instruments designed to assess person-centered care, most have focused only on some dimensions of the Mead & Bower model [4]. In a meta-analysis that examined instruments focused on specific dimensions of person-centered care, Pascual and colleagues [15] identified five questionnaires focused on the dimension “patient as a person” or “biopsychosocial perspective”. Fifteen questionnaires focus on the dimension “sharing power and responsibility,” and nineteen focus on the “therapeutic alliance” dimension. It is noteworthy that 65% of these instruments were validated in the United States and the United Kingdom, with 80% in English-speaking countries. Only 7.7% of the analyzed questionnaires were validated in Spanish, but none were focused on the Mexican population. These investigators conducted an analysis based on the COSMIN scale (Consensus-based Standards for the selection of health Measurement Instruments) [16], which measures the quality and design of instruments, detecting methodological issues.

Thus, the aim of the present study is to design and validate an instrument that assesses the extent to which PEMEX (Petróleos Mexicanos) healthcare services provide person-centered care according to user perceptions. In addition to employing a suitable methodology that enables instrument generalization, the design will be based on the four dimensions of the person-centered care model [4]. The purpose of this work is to apply an instrument to guide the practice of healthcare professionals, enabling them to attain the benefits of patient empowerment, facilitating shared responsibilities in healthcare, and enhancing the quality of medical care.

## Methods

**Fig. 1:**
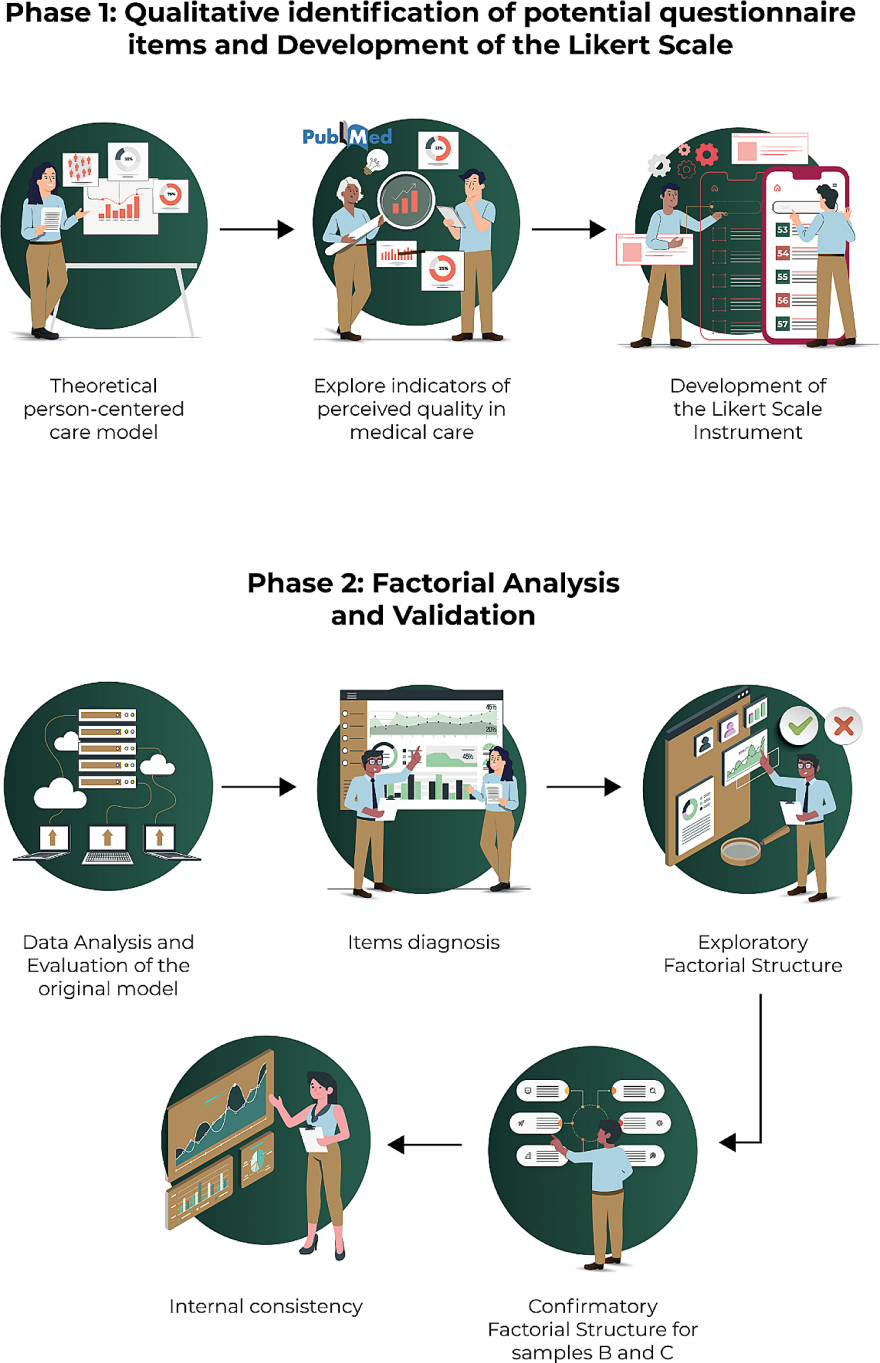
Design and validation of the Person-Centered Care Instrument

The present is an instrumental study [17], included in the category of studies that analyze the psychometric properties of psychological measurement instruments, including two phases (Fig. 1). In Phase 1, the instrument items were developed following the application of a questionnaire to a group of PEMEX healthcare service users on the care received. It is worth noting that the questionnaire was based on the dimensions of the Person-Centered Care Method [4]. Phase 2 involved the validation of the Person-Centered Care Instrument in Health Services.

### Phase 1: Qualitative identification of potential questionnaire items and development of the Likert Scale

#### Participants

The participants consisted of 30 adult voluntary individuals [18], both men and women, who were users of PEMEX healthcare services at Mexico City, Mexico. Inclusion criteria required signing informed consent. The sole exclusion criterion was not completing the questionnaire.

#### Instrument

To explore indicators of perceived quality in medical care by PEMEX health users, an open questionnaire consisting of 9 questions was used, which was validated based on the agreement between 4 expert judges [19, 20]. This questionnaire was applied by the researcher to the 30 enrolled participants. The sample size was based on the principle of theoretical saturation contemplated by qualitative research [21], and a number of participants similar to that of previous studies was used [18]. The questions were grouped according to the dimensions of the theoretical person-centered care model: (1) patient as a person, (2) biopsychosocial perspective, (3) therapeutic alliance, and (4) sharing power and responsibility [4]. (Appendix C)

#### Procedure

Participants were recruited by investigators at the Medical Unit of Petróleos Mexicanos and the Central South Hospital, which are advertised to PEMEX. Subjects voluntarily responded to the questionnaire in August 2022. Emphasis was placed on confidentiality and personal data protection. After data collection, a content analysis was conducted to identify positive and negative indicators for each dimension, which collectively were the basis for a first Likert scale instrument with 71 items.

#### Development of the Likert Scale instrument

The scale items were evaluated by four experts in the field using the following criteria: clarity, wording, coherence, and relevance to each dimension. Based on expert opinions and due to a lack of congruence and relevance to the respective dimensions, 14 items were removed, resulting in 57 items: 19 for “patient as a person”, 13 for “biopsychosocial perspective,” 13 for “therapeutic alliance,” and 12 for the “sharing power and responsibility” dimension. Please, see the first instrument version in Appendix B.

### Phase 2: Factorial analysis and validation

At the beginning of phase 2, a confirmatory factor analysis was performed including the 4 factors of Mead & Bower’s person-centered care model [4]. Inadequate fit indices or high correlations between factors implied an exploratory factor analysis to determine the initial structure of the instrument and a confirmatory factor analysis. In addition, the internal consistency of the instrument was assessed to determine the capability to measure person-centered care in a sample of Pemex healthcare service users.

#### Participants

Three groups of participants were recruited using a nonrandom convenience sampling technique. Sample A included 330 participants aged between 18 and 97 years (M = 51.38; SD = 15.57), 208 (63%) females and 122 (37%) males. Sample B consisted of 335 participants aged between 18 and 93 years (M = 51.06; SD = 14.13), 204 (60.9%) females and 131 (39.1%) males. Sample C included 130 participants aged between 18 and 88 years (M = 50.19; SD = 13.85), 89 (68.5%) females and 41 (31.5%) males. The personnel of the Health Services Quality Department of Petróleos Mexicanos invited users to participate and self-administer the instrument during the visit to any of Pemex’s medical facilities, including central and regional hospitals, clinics, and offices.

The confirmatory factor analysis was performed using the Group B to corroborate the 4-factor model, exceeding a minimum of 100 to 150 participants contemplated for SEM (structural equation modeling) models [22]. Given high correlations between factors, an overfactoring effect and a risk of overfitting [23], an exploratory factor analysis was carried out with this same group, assigning 5 participants per item, thus fulfilling with the minimum sample as previously described [24]. On the other hand, Groups B and C met the criteria required for conducting confirmatory factorial analysis [25] and the sample required to perform the confirmatory factorial analysis [22]. The inclusion criteria for all groups were the signing of informed consent and being adults (> 18 years); meanwhile, subjects who did not answer the instrument questions were excluded.

### Instruments

A Likert scale was employed to evaluate the quality of health care, consisting of 57 items with 6 response options ranging from 1 (completely disagree) to 6 (completely agree). Items were divided into four dimensions: (1) Patient as a person; (2) Biopsychosocial perspective; (3) Therapeutic alliance; and (4) Sharing power and responsibility. The scale was scored by the arithmetic sum of each item’s score, with higher scores indicating higher quality of health care.

#### Design

Instrumental [17]. The construct validity of the scale was assessed through a factorial analysis, and internal consistency was assessed by using Cronbach’s α and McDonald’s ω.

#### Procedure

Samples A and B completed the instrument in November 2022, while Sample C completed the instrument in February 2023. Subjects were requested for voluntary participation by investigators who explained the study objectives and addressed any questions. Subjects eligible for being enrolled in the study signed an informed consent form and then answered the instrument.

### Data analysis

Data were analyzed in four sequential steps. Step 1: the structure of the 4-factor model described by Mead & Bower model [4] was evaluated through a confirmatory factor analysis in the Group A. Step 2: Given high correlation between factors resulting from the evaluation of the 4-factor model, it was decided to start with an exploratory factor analysis to identify the initial structure of the instrument using data from sample A. Step 3: Here, data from Sample A were used to identify the initial structure of the instrument through exploratory factorial analysis. Step 2: The investigators used Sample B to verify the previously assessed instrument structure by confirmatory factorial analysis. In Step 4, confirmatory factorial analysis was performed using Sample C. This additional confirmatory factorial analysis was performed due to the number of refinements on the adjusted model during the previous steps.

In step 1, where the 4-factor model was evaluated to verify the goodness of fit of the model, the following indices were addressed: for absolute fit, the chi-square test (χ2) and the chi-square divided by degrees of freedom (χ2/df); for a parsimonious fit, the root mean square error of approximation (RMSEA) with the confidence interval (CI); and for incremental fit, the Tucker-Lewis index (TLI) and the comparative fit index (CFI). Acceptable fit values were considered as follows: χ2/df ≤ 5, RMSEA ≤ 0.08, TLI ≥ 0.90, CFI ≥ 0.90; and excellent fit values: χ2/df ≤ 2, RMSEA ≤ 0.05, TLI ≥ 0.95, CFI ≥ 0.95 [26, 27]; Likewise, the presence of independent factors was evaluated (i.e., r 0.80), which would allow an overfactoring effect to be avoided [23].

Afterward, an item analysis was performed, which involved identifying extreme responses within 90% or more of the choices, following this item elimination. The mean, standard deviation, and skewness and kurtosis coefficients were calculated. Univariate normality was assessed using the Shapiro‒Wilk test, while multivariate normality was assessed using the Mardia coefficient. Discriminative power for items was estimated using the extreme groups strategy, contrasting scores below the first quartile and above the third quartile of each item with one-tailed independent groups t tests. This test was selected due to its robustness and capability to handle deviations from normality [28]. The corrected item-total correlation was also calculated. Items with tests lacking discriminative capability or r values < 0.20 were removed [29]. To detect redundant items, a multicollinearity analysis was performed, and items with an interitem correlation of ≥ 0.80 were removed [30].

By correlation matrix determinant calculation, Bartlett’s test, and the Kaiser‒Meyer‒Olkin (KMO) index along with the confidence interval (CI), the sample adequacy of the data was assessed. In cases of inadequate KMO values (i.e., KMO ≤ 0.70), an individual adequacy analysis was performed based on the anti-image correlation matrix (AIM), and the item with the lowest value on the AIM’s main diagonal was eliminated. Following this, the sample adequacy tests were recalculated.

To mitigate factor overestimation, a parallel analysis was conducted, retaining factors that explained variance exceeding the 95th percentile random-generated factors. This strategy provides objective grounds for factor retention [31] as opposed to methods such as Kaiser’s criterion or scree plot analysis, which often overestimate the number of factors [32].

Subsequently, an exploratory factorial analysis was conducted using the polychoric correlation matrix by the robust diagonally weighted least squares (DWLS) method and oblique promax rotation. Retaining a factor required a minimum of 3 items, each with a factor loading ≥ 0.40 (i.e., simple factorial structure), a communality (h2) ≥ 0.32 [33], conceptual congruence between item and factor, and each factor showing a reliability ≥ 0.70 as calculated by Cronbach’s α and McDonald’s ω.

In Step 3, a confirmatory factorial analysis was performed using the maximum likelihood estimation method. The same criteria using in the step 1 were used to verify the goodness of fit of the model. During this process, both statistical criteria (modification indices and factor loading of each item) and theoretical considerations (conceptual coherence of item and factor) were considered to maintain the instrument’s conceptual value [34]. Internal consistency was evaluated using the final adjusted model. Items with a Cronbach’s α and McDonald’s ω > 0.94 were assessed for multicollinearity, and items with an interitem correlation ≥ 0.80 were removed.

Step 4 included a confirmatory factorial analysis and a multigroup confirmatory factorial analysis, both using the maximum likelihood method.

Data analysis was performed using SPSS v.23, AMOS v.21, and FACTOR v.12.03.02. When applicable, a significance level of *p* ≤ .05 was considered significant.

## Results

### Original model evaluation

Table 1 shows the results of the initial factorial model adjustment. Acceptable adjustment criteria were obtained by χ2/gl and RMSEA indices. Although the CFI and the TLI remain below the acceptable level, re-specification actions based on the analysis of the modification indices and the theoretical conception could achieve an acceptable model. However, the high correlations between the 4 factors (i.e., *r* ≥ .80) (Table 2) indicate an overfactoring effect and therefore the risk of overfitting [23] increases. Furthermore, these values suggest an uneconomical theoretical model, since with a smaller number of factors and/or reagents a more parsimonious instrument could be obtained. Due to this, an exhaustive analysis of the instrument was carried out, starting with the evaluation of each individual item behavior, and conducting an exploratory and confirmatory factor analysis.

**Table 1:** Adjustment criteria for the confirmatory factor structure of the initial instrument (57 items and 4 factors)

	χ2	χ2/gl	RMSEA	IC RMSEA	TLI	CFI
**Model**	7373.73	4.81	0.076	0.074 a 0.077	0.85	0.85

**Table 2:** Correlation between factors of the initial instrument

	F1	F2	F3	F4
F1	1			
F2	0.89	1		
F3	0.87	0.96	1	
F4	0.85	0.93	0.95	1

### Items diagnosis

No item concentrated ≥ 90% of choices in any of the extreme response options. The mean and standard deviation of item scores ranged from 3.76 to 5.19 and from 0.86 to 1.62, respectively. The skewness and kurtosis coefficients were ≤ |1.11| and ≤ |1.65|, respectively, with no evidence of univariate normality (*p* < .001) or multivariate normality (*p* < .001). All scale items discriminated (*p* < .05). Most items showed a corrected item-total correlation > 0.30, except for items 22, 31, and 56 (Table 3). Interitem correlation analysis identified an *r* ≥ .80 in 15 items, which were removed for subsequent analyses (i.e., 4, 5, 6, 20, 21, 27, 29, 35, 36, 38, 39, 41, 43, 45, 49).

**Table 3:** Descriptive analysis of instrument items to evaluate person-centered care in health services

Item	M	SD	S	C	rr-tc	Item	M	SD	S	C	rr-tc	Item	M			SD		S		C		rr-tc	
**1**	5.19	0.97	-1.11	1.57	0.69	**21**	4.90	0.93	-0.24	-0.46	0.85	**41**	4.92			0.89		-0.07		-1.14		0.88	
**2**	5.08	1.00	-0.99	1.30	0.73	**22**	4.09	1.62	-0.38	-0.87	0.29	**42**	4.90			0.92		-0.19		-0.78		0.88	
**3**	4.99	1.01	-0.77	0.67	0.76	**23**	4.56	1.22	-0.68	0.33	0.46	**43**	4.88			0.96		-0.44		0.23		0.86	
**4**	5.04	0.94	-0.52	-0.27	0.81	**24**	4.36	1.15	-0.48	0.63	0.46	**44**	4.88			0.88		0.04		-1.22		0.81	
**5**	5.03	0.93	-0.41	-0.57	0.80	**25**	4.90	0.99	-0.55	0.26	0.72	**45**	4.89			0.87		-0.05		-1.05		0.87	
**6**	4.88	0.97	-0.24	-0.63	0.79	**26**	4.49	1.21	-0.62	0.40	0.52	**46**	4.89			0.90		-0.07		-1.01		0.80	
**7**	4.95	0.96	-0.44	-0.21	0.82	**27**	4.79	0.94	-0.14	-0.56	0.84	**47**	5.00			0.88		-0.03		-1.65		0.78	
**8**	4.81	1.04	-0.51	0.40	0.76	**28**	4.62	1.01	-0.34	0.40	0.73	**48**	4.92			0.94		-0.22		-0.99		0.74	
**9**	4.47	1.45	-0.60	-0.55	0.47	**29**	4.88	0.92	-0.23	-0.38	0.84	**49**	4.98			0.88		-0.12		-1.22		0.85	
**10**	4.93	0.99	-0.49	-0.15	0.79	**30**	4.86	0.92	-0.13	-0.80	0.85	**50**	4.90			0.96		-0.27		-0.56		0.71	
**11**	4.68	1.11	-0.54	0.06	0.65	**31**	3.76	1.48	-0.18	-0.61	0.25	**51**	4.85			0.90		0.05		-1.21		0.71	
**12**	4.62	1.13	-0.59	0.44	0.65	**32**	4.43	1.18	-0.80	1.18	0.63	**52**	4.76			1.01		-0.35		0.19		0.72	
**13**	4.61	1.09	-0.41	0.18	0.65	**33**	4.71	1.07	-0.74	1.13	0.78	**53**	4.91			0.90		-0.02		-1.37		0.86	
**14**	4.99	0.94	-0.44	-0.35	0.79	**34**	4.87	0.93	-0.36	0.28	0.86	**54**	4.91			0.94		-0.15		-0.77		0.73	
**15**	4.43	1.19	-0.61	0.66	0.46	**35**	4.88	0.86	0.03	-1.13	0.89	**55**	5.04			0.90		-0.13		-1.65		0.86	
**16**	4.10	1.33	-0.51	0.13	0.49	**36**	4.93	0.88	-0.10	-1.08	0.85	**56**	4.06			1.53		-0.29		-0.76		0.12	
**17**	4.49	1.23	-0.67	0.54	0.55	**37**	4.96	0.88	-0.09	-1.27	0.85	**57**	4.71			1.03		-0.48		0.61		0.60	
**18**	4.68	1.03	-0.41	0.47	0.71	**38**	4.91	0.91	-0.40	0.05	0.87												
**19**	4.79	0.98	-0.23	-0.56	0.80	**39**	4.92	0.91	-0.17	-0.96	0.87												
**20**	4.99	0.94	-0.44	-0.38	0.83	**40**	4.85	0.93	-0.25	-0.32	0.84												

M: mean; SD: standard deviation; S: skewness; K: kurtosis; item-total corr.: corrected item-total correlation.

### Exploratory factorial structure

The polychoric matrix was initially nonpositive definite, so the sweet smoothing algorithms developed by [35] were implemented. Once the matrix was positive definite, tests of sample adequacy indicated that scale data were appropriate for conducting the factor analysis: KMO = 0.97, Bartlett’s test *p* < .01, and determinant of the correlation matrix < 0.000001. Parallel analysis identified a single factor that explained the variance exceeding the 95th percentile of variance by random factors. The exploratory factor analysis retained 35 items (Table 4), which met the previously specified criteria, organized into a single factor that explained 49% of the variance with an internal consistency α Cronbach and ω McDonald = 0.97. Items 9, 13, 48, and 51 were removed due to communality < 0.32.

**Table 4:** Exploratory Factorial Analysis of the Instrument to Evaluate Person-Centered Care in Health Services

*R*	F	rr-tc	h2	*R*	F	rr-tc	h2	*R*	F	rr-tc	h2
**1**	0.80	0.68	0.65	**17**	0.64	0.57	0.41	**40**	0.90	0.83	0.80
**2**	0.58	0.72	0.34	**18**	0.79	0.72	0.63	**42**	0.67	0.87	0.45
**3**	0.60	0.75	0.36	**19**	0.86	0.80	0.74	**44**	0.86	0.80	0.73
**7**	0.92	0.82	0.84	**23**	0.59	0.46	0.35	**46**	0.87	0.79	0.75
**8**	0.59	0.76	0.35	**24**	0.57	0.48	0.32	**47**	0.59	0.76	0.35
**9**	0.52	0.44	0.27	**25**	0.57	0.72	0.32	**48**	0.56	0.73	0.31
**10**	0.61	0.78	0.38	**26**	0.61	0.54	0.37	**50**	0.76	0.70	0.57
**11**	0.71	0.66	0.51	**28**	0.80	0.73	0.64	**51**	0.54	0.70	0.30
**12**	0.72	0.65	0.52	**30**	0.91	0.84	0.83	**52**	0.57	0.72	0.32
**13**	0.52	0.67	0.27	**32**	0.75	0.64	0.56	**53**	0.65	0.85	0.43
**14**	0.61	0.78	0.37	**33**	0.62	0.78	0.38	**54**	0.56	0.72	0.32
**15**	0.58	0.48	0.34	**34**	0.66	0.85	0.43	**55**	0.94	0.85	0.87
**16**	0.57	0.51	0.33	**37**	0.65	0.84	0.42	**57**	0.71	0.61	0.50

R: item; F: factor; item-total corr. : corrected item-total correlation; h2: communality

### Confirmatory factorial structure, sample B

The initial evaluation of factor structure was unsatisfactory, prompting a review of the modification indices aimed at model refinement to achieve at least acceptable fit indices. The analysis focused on the covariance value between items. With high values observed between item pairs, their conceptual relevance and wording were scrutinized. When items had similar semantic structures (e.g., “I receive respectful treatment at this medical unit” and “The rules and regulations are communicated and respected at the medical unit”) or referred to similar ideas (e.g., “There is good communication between doctors and me” and “The doctors’ good communication in this unit makes me feel committed to my health”), the item with the lower factor loading was removed. This process led to the removal of 24/35 items (1, 3, 7, 11, 12, 16, 17, 23, 24, 26, 28, 30, 33, 34, 37, 40, 42, 44, 47, 50, 52, 53, 54, 55), achieving acceptable (χ2/df, RMSEA) to high (TLI, CFI) fit criteria (Table 5). Sample B showed internal consistency of the 11-item model, as shown by Cronbach’s α and McDonald’s ω = 0.93.

**Table 5:** Fit Criteria for Confirmatory Factorial Structure of the Instrument to Evaluate Person-Centered Care in Healthcare Services (Sample B)

Model	χ2	χ2/gl	RMSEA	IC RMSEA	TLI	CFI
**Initial**	2659.28	4.74	0.10	0.10 a 0.11	0.80	0.81
**Final**	127.53	2.89	0.07	0.06 a 0.09	0.95	0.96

### Confirmatory factorial structure, sample C

**Fig. 2:**
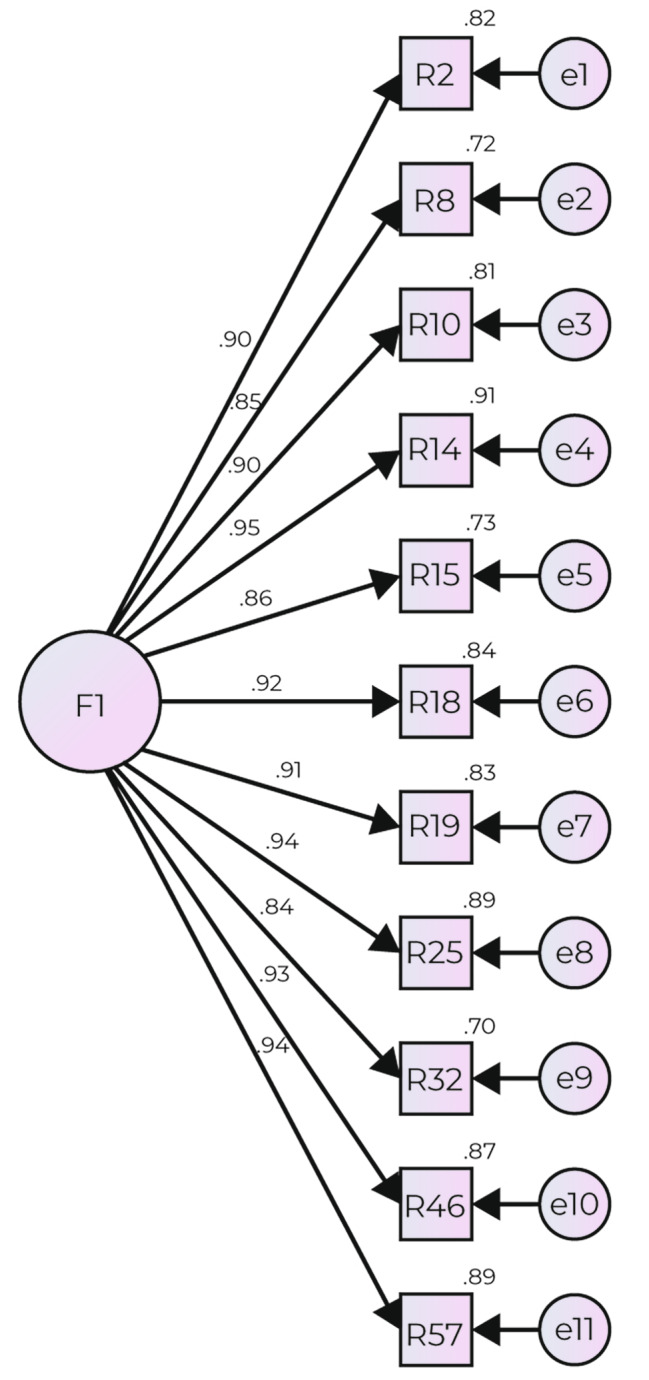
Factorial structure of the instrument to evaluate Person-Centered Care in Health Services. Values shown on the lines that join the factor with the reactants represent the factor loadings and those values placed on one side of the rectangle that represents the reactants are the R2. Hair et al. [36] argues that standardized factor loadings should ideally be 0.7, which implies a variance of 49%, that is, R2 = 0.49

Table 6 shows the results of the factorial model fit with 11 resulting items from Sample C (Fig. 2). Acceptable fit criteria are observed for χ2/gl, TLI, and CFI indices; however, the RMSEA index value was not acceptable.

**Table 6:** Goodness of Fit Criteria for the Confirmatory Factorial Structure of the Instrument to Evaluate Patient-Centered Care in Health Services (Sample B)

	χ2	χ2/gl	RMSEA	IC RMSEA	TLI	CFI
**Model**	151.44	3.43	0.13	0.11 a 0.16	0.93	0.94

The internal consistency of the 11-item model using Sample C was Cronbach’s α and McDonald’s ω = 0.98.

The final items of the instrument are shown in Table 7, indicating to which factor of the original instrument they belonged (Appendix C).

**Table 7:** Final instrument items to evaluate Person-Centered Care in Health Services

	Item	Factor
2	During the medical consultation, I feel comfortable with the doctor’s comments.	Biopsychosocial perspective
8	When I’m expressing myself, the doctor looks me in the eyes.	Biopsychosocial perspective
10	The treatment from doctors in this institution is friendly.	Biopsychosocial perspective
14	I have received good care at this medical unit.	Biopsychosocial perspective
15	In this medical unit, the furniture intended for patient use is comfortable and functional.	Biopsychosocial perspective
18	Doctors take my daily activities into account when instructing me about the type of diet I should follow.	Biopsychosocial perspective
19	In the medical unit, doctors collaborate with colleagues from other areas as a team to reach the correct diagnosis.	Biopsychosocial perspective
25	This medical unit maintains sanitized and clean spaces to provide proper medical care.	Patient as a person
32	At the medical unit, they respect the assigned schedule for my medical care.	Patient as a person
46	I have the power to decide on the management of my health once the doctor explains the treatment alternatives to me.	Sharing power and responsibilities
57	During medical care, when I feel uncomfortable, I express it to the healthcare staff.	Sharing power and responsibilities

## Discussion

Patients’ positive experiences regarding medical care and low complication rates have been proposed as essential components of healthcare quality, encompassing patient-centered care. While some authors debate whether there is a correlation between technical care quality and interpersonal quality as reflected by patient satisfaction [37], facts have shown that reducing complications can lead to a better hospital experience. On the other hand, considering and addressing individuals’ needs promotes shared responsibility and increases success in health care, creating a virtuous circle that encompasses both technical and interpersonal aspects of quality. Hence, it is important to measure patient-centered care.

The instrument validation based on exploratory factorial analysis indicated a unifactorial structure with high overall internal consistency, explaining 49% of the variance.

The single-factor model is consistent with the fact that original model showed redundancy between factors [23]. In this way, with a smaller number of factors and items, redundant elements were eliminated, and a parsimonious instrument was obtained, adjusted from statistical estimates and a theoretical conception derived from the Patient-Centered Care model of Mead and Bower [4].

The inconsistency between the instrument structure and the conceptual framework (Mead and Bower’s Patient-Centered Care model [4]), which identifies four dimensions: (1) biopsychosocial perspective, (2) patient as a person, (3) therapeutic alliance, and (4) shared power and responsibilities might be supported by the close conceptual relationship among the dimensions considered in the model. For example, the “biopsychosocial perspective” of care implies healthcare staff’s willingness to engage with the range of difficulties raised by patients, not only with biomedical issues [38]. This is closely related to the concept of the “patient as a person” or individual experience illness as described by Armstrong [39], encompassing the difficulties expressed by patients beyond the biomedical issues. On the other hand, Balint [40] notes that healthcare providers should understand signs and symptoms not only in terms of diseases but also as expressions of the patient’s individuality, conflicts, and problems. This suggestion is in accordance with the “biopsychosocial factor” tightly related to the dimension “patient as a person”. On the other hand, the factor “shared power and responsibility”, described by Byrne and Long [41], refers to patients’ involvement in their health care and implies a symmetrical relationship, in line with what the “biopsychosocial” and “patient as a person” factors imply; this involves recognizing patients’ needs and preferences to encourage them to express ideas, listen, reflect, and collaborate. The quality of the doctor‒patient relationship, considering the above discussion, leads to the so-called “therapeutic alliance,” a factor that involves shared management of illness and decision-making. Crow et al. [42] point out that friendly and understanding attitudes from the patient improve their conditions, as affection impacts the outcome of medical treatment-mediated health. Thus, the selected items generally encompass the concept of patient-centered care, where factors beyond the biomedical model are considered, focusing on patients’ individualities and fostering an affectionate and equal relationship between doctor and patient. In summary, the abovementioned factors suggest that exploratory structure is conceptually and empirically consistent.

The parallel analysis carried out for exploratory factor analysis provides this study with assurance on the instrument factorial structure [31]. Furthermore, the use of oblique rotation increases the likelihood that psychological constructs are correlated with each other, as opposed to an orthogonal case [43]. Additionally, employing confirmatory factorial analysis allowed us to evaluate the initial identified structure more strictly, enabling the model to be respecified until achieving at least acceptable fit indices. Model refinement involved the removal of items based on high covariance between pairs with similar semantic structures. This procedure, together with the establishment of fit criteria ranging from acceptable to high, resulted in a brief instrument with high internal consistency (Cronbach’s alpha = 0.93).

Finally, a new confirmatory factorial analysis was performed with the 11-item scale after the phase 2 refinement, achieving adequate goodness-of-fit criteria on most of the considered items, thus confirming the previously proposed model. The sample size in the final confirmatory factor analysis could affect the values obtained by RMSEA. According to Morata-Ramírez [44], the percentage of acceptance of the models through RMSEA increases as the sample size increases, with mean values of 0.133 for samples of 100 subjects decreasing to 0.043 for samples of 850 subjects. An unacceptable RMSEA indicates that there may be an inconsistency between the estimated theoretical model and the real behavior of the populations, suggesting the evaluation and/or the review of the items wording or the re-specification of the model itself. However, the suggested cut-off points such as RMSEA, CFI and TLI are largely based on intuition and experience rather than statistical justification [45]. In this regard, although Hu and Bentler [46] suggested a RMSEA less than 0.06 and a CFI and TLI greater than 0.95, these authors point out that such criteria only refer to continuous data that are analyzed using the maximum likelihood (ML) of the normal theory. These authors warn that the suggested cut-off values may not generalize to conditions that were not addressed in their study, nor to estimation methods other than ML.

One of the study limitations is that only patients from PEMEX healthcare service users were considered, making it difficult to generalize to other populations. Furthermore, other types of validity testing are needed, such as discriminant and predictive validation. Future studies should address these limitations.

The application of an instrument that measures the level of person-centered care allows for interventions or strategies to improve patient engagement during care and to optimize healthcare resources [47]. In this regard, Holt [12] points out that improving the quality of healthcare should involve understanding the nature and relationships between patient experience, history, and expectations. The instrument we designed and validated should be used for ongoing and systematic assessment, identifying the degree to which the care provided is patient-centered. When healthcare services fail to meet patients’ expectations of care, it is less likely for patients to engage responsibly, adhere to treatments, and share responsibility for their own health condition with physicians and healthcare institutions [48].

## Conclusions

The instrument, validated with users of PEMEX healthcare services, demonstrates adequate psychometric properties to be used as a tool for measuring the degree of person-centered care. The use of this instrument could represent a strategy or means for promoting an innovative and functional healthcare model that encompasses both the interpersonal and technical dimensions, thus moving away from the exclusively biomedical model.

### Electronic supplementary material

Below is the link to the electronic supplementary material.

Supplementary Material 1

Supplementary Material 2

Supplementary Material 3

## Data Availability

and Materials. The datasets of the current study are available from the corresponding author upon reasonable request.

## Abbreviations

MAS/ACM: Anti-image Correlation Matrix
CFI: Comparative Fit Index
CI: Confidence Interval
COSMIN: Consensus-based Standards for the selection of health Measurement Instruments
DWLS: Diagonally weighted least squares
KMO: Kaiser-Meyer‐Olkin
C/K: Kurtosis
M: Mean
PEMEX: Petróleos Mexicanos
rr-tc/ ri-tc: Correction of item-total correlations
RMSEA: Root mean square error of approximation
S: Skewness
SD: Standard Deviation
TLI: Tucker‒Lewis Index
S: Skewness
