# Supplementary material for: Design and validation of an instrument to evaluate Person-Centered care in health services
Source: Arch Public Health. 2024 Aug 14;82:123. doi: 10.1186/s13690-024-01324-2 (PMC11323455; doi:10.1186/s13690-024-01324-2)
Supplement: Supplementary file 1 — Supplementary Material 1 [file 13690_2024_1324_MOESM1_ESM.docx]

**Appendix C**

**Validated final instrument.**

**Patient-centered attention instrument**

This scale aims to identify the extent to which you receive “patient-centered” medical care.

Instructions. Choose the appropriate answer option for each statement according to your experience.

| Items | Totally disagree | Strongly disagree | In disagreement | Agree | Strongly agree | Totally agree |
| --- | --- | --- | --- | --- | --- | --- |
| 1. During the medical consultation, I feel comfortable with the doctor's comments. |  |  |  |  |  |  |
| 1. When I'm expressing myself, the doctor looks me in the eyes. |  |  |  |  |  |  |
| 1. The treatment from doctors in this institution is friendly. |  |  |  |  |  |  |
| 1. I have received good care in this medical unit. |  |  |  |  |  |  |
| 1. The furniture intended for patient use in this medical unit is comfortable and functional. |  |  |  |  |  |  |
| 1. Doctors consider my daily activities when instructing me about the type of diet I should follow. |  |  |  |  |  |  |
| 1. In the medical unit, doctors collaborate with colleagues from other areas to reach the correct diagnosis. |  |  |  |  |  |  |
| 1. This medical unit maintains sanitized and clean spaces to provide proper medical care. |  |  |  |  |  |  |
| 1. At the medical unit, they respect the assigned schedule for my medical care. |  |  |  |  |  |  |
| 1. I have the power to decide on the management of my health once the doctor explains the treatment alternatives to me. |  |  |  |  |  |  |
| 1. When I feel uncomfortable during medical care, I express it to the healthcare staff. |  |  |  |  |  |  |
