# Supplementary material for: Design and validation of an instrument to evaluate Person-Centered care in health services
Source: Arch Public Health. 2024 Aug 14;82:123. doi: 10.1186/s13690-024-01324-2 (PMC11323455; doi:10.1186/s13690-024-01324-2)
Supplement: Supplementary file 2 — Supplementary Material 2 [file 13690_2024_1324_MOESM2_ESM.docx]

**Appendix B**

**Initial Instrument Reagents**

1. In this medical unit, I receive respectful treatment.
2. During the medical consultation, I feel comfortable with the doctor's comments.
3. Over time, the doctor arrived at an appropriate diagnosis and treatment.
4. The doctor pays attention to me when I explain why I visit the medical unit.
5. The doctor pays attention to me when I am talking.
6. When I show my emotions during the consultation, the doctor is interested in them.
7. During the consultation, the doctor focuses on me.
8. When I'm expressing myself, the doctor looks at me in the eyes.
9. The doctor's treatment of the patient in this medical unit is disinterested (cold)
10. The treatment from doctors in this institution is friendly.
11. In this medical unit, they care about respecting office hours.
12. Health personnel should consider my activities at home/work when scheduling an appointment.
13. The health staff is friendly even when I have difficulty getting to my appointment on time.
14. I have received good care in this medical unit.
15. The furniture intended for patient use in this medical unit is comfortable and functional.
16. If I need an appointment with specialists for the first time, they provide it to me in less than two weeks.
17. If I require imaging studies (x-rays, ultrasound, CT scans, MRIs), they schedule me at least 15 days before my appointment with the specialist.
18. Doctors consider my daily activities when instructing me about the type of diet I should follow.
19. Doctors collaborate with colleagues from other areas in the medical unit to reach the correct diagnosis.
20. The health personnel always address me respectfully when providing me with the required information.
21. The health personnel care that I understand their instructions.
22. The health personnel are indifferent during the care of my illness.
23. During my physical examination, the medical staff considered situations that may make me feel uncomfortable.
24. The staff of this medical unit solves maintenance problems.
25. This medical unit maintains sanitized and clean spaces to provide proper medical care.
26. This medical unit has all the medicine I need.
27. The doctor considers my daily activities when giving me treatment and recommendations.
28. Doctors consider the times I have to eat in the dietary recommendations they give me.
29. Health personnel encourage me when I feel nervous about a procedure.
30. Doctors are understanding when I don't feel ready for a procedure.
31. On most occasions, I wait more than an hour to receive medical attention in an outpatient clinic.
32. At the medical unit, they respect the assigned schedule for my medical care.
33. The medical staff constantly communicates my health status to me.
34. There is good communication between the doctors and me.
35. When I have doubts, I tell the doctor, and he solves them.
36. The doctors are clear and precise with instructions about my procedures, diagnoses, and treatments.
37. The medical unit staff is honest with the information they give me about my health status.
38. When the doctors gave me the diagnosis, they gave me confidence in this certainty.
39. I feel confident about the decisions doctors make in my health care.
40. The doctor takes my opinion into account when treating my illness.
41. The doctor explains his instructions to me until I understand it
42. The doctors pay attention when I explain what is happening to me.
43. When I express my thoughts and beliefs, health personnel respect them.
44. I participate with the doctor to have good management of my illness.
45. The doctor's explanation about my illness is easy to understand.
46. I have the power to decide on the management of my health once the doctor explains the treatment alternatives to me.
47. I am aware of my responsibility to comply with medical instructions.
48. The doctor and I share responsibility for my health care.
49. The clarity with which the doctors explain their instructions to me allows me to follow them to the letter.
50. When I am talking about my illness for the first time, I try to be clear so that the doctor can easily understand me.
51. When doctors give me instructions, I stick to them.
52. In the medical unit, the regulations are made known and respected.
53. The good communication of the doctors in this unit makes me feel committed to my health.
54. During medical care, I ask if the doctor's explanation is unclear.
55. The doctor always speaks to me with the truth, even if it is terrible news about my health.
56. Occasionally, I have hidden information from doctors, causing failures in my medical care.
57. When I feel uncomfortable during medical care, I express it to the healthcare staff.
