# Supplementary material for: Design and validation of an instrument to evaluate Person-Centered care in health services
Source: Arch Public Health. 2024 Aug 14;82:123. doi: 10.1186/s13690-024-01324-2 (PMC11323455; doi:10.1186/s13690-024-01324-2)
Supplement: Supplementary file 3 — Supplementary Material 3 [file 13690_2024_1324_MOESM3_ESM.docx]

**Appendix A**

Open questions for item construction

| Patient as a person | 1. What circumstances make you feel that your needs have been met when you receive medical care? |
| --- | --- |
|  | 1. What considerations should doctors make to understand you as a patient better? |
| Biopsychosocial perspective | 1. What emotions or feelings should be taken into account during the medical consultation? |
|  | 1. How could we help you meet your needs during your health care? |
|  | 1. What aspects of your style and lifestyle are essential during medical care? |
| Therapeutic alliance | 1. What must the doctor and the patient do for the doctor-patient relationship to work and the treatment to be successful? |
|  | 1. What does an excellent doctor-patient relationship mean to you? |
| Share power and responsibility | 1. In what specific situation do you consider that you have the power to decide about your health and your treatment? |
|  | 1. In medical care, what is the patient's and doctor's responsibility so that you have successful treatment? |
